# Cellulases without carbohydrate-binding modules in high consistency ethanol production process

**DOI:** 10.1186/1754-6834-7-27

**Published:** 2014-02-21

**Authors:** Annukka Pakarinen, Mai Østergaard Haven, Demi Tristan Djajadi, Anikó Várnai, Terhi Puranen, Liisa Viikari

**Affiliations:** 1 Department of Food and Environmental Sciences, University of Helsinki, PO 27, 00014 Helsinki, Finland; 2 DONG Energy A/S, Kraftværksvej 53, 7000 Fredericia, Denmark; 3 Department of Chemistry, Biotechnology and Food Science, Norwegian University of Life Sciences, PO Box 5003, N-1432 Aas, Norway; 4 Roal Oy, Tykkimäentie 15, FIN-05200 Rajamäki, Finland

**Keywords:** Carbohydrate-binding modules, CBM, Cellulases, Cellobiohydrolases, Recyclability, Lignocellulose, Hydrolysis, High consistency

## Abstract

**Background:**

Enzymes still comprise a major part of ethanol production costs from lignocellulose raw materials. Irreversible binding of enzymes to the residual substrate prevents their reuse and no efficient methods for recycling of enzymes have so far been presented. Cellulases without a carbohydrate-binding module (CBM) have been found to act efficiently at high substrate consistencies and to remain non-bound after the hydrolysis.

**Results:**

High hydrolysis yields could be obtained with thermostable enzymes of *Thermoascus aurantiacus* containing only two main cellulases: cellobiohydrolase I (CBH I), Cel7A and endoglucanase II (EG II), Cel5A. The yields were decreased by only about 10% when using these cellulases without CBM. A major part of enzymes lacking CBM was non-bound during the most active stage of hydrolysis and in spite of this, produced high sugar yields. Complementation of the two cellulases lacking CBM with CBH II (*Ct*Cel6A) improved the hydrolysis. Cellulases without CBM were more sensitive during exposure to high ethanol concentration than the enzymes containing CBM. Enzymes lacking CBM could be efficiently reused leading to a sugar yield of 90% of that with fresh enzymes. The applicability of cellulases without CBM was confirmed under industrial ethanol production conditions at high (25% dry matter (DM)) consistency.

**Conclusions:**

The results clearly show that cellulases without CBM can be successfully used in the hydrolysis of lignocellulose at high consistency, and that this approach could provide new means for better recyclability of enzymes. This paper provides new insight into the efficient action of CBM-lacking cellulases. The relationship of binding and action of cellulases without CBM at high DM consistency should, however, be studied in more detail.

## Background

Efficient enzymatic hydrolysis of lignocellulosic plant cell walls to platform sugars is a key process in all future biotechnical biomass conversion processes to fuels or chemicals. Although a significant reduction in enzyme production costs has been reported by the major enzyme producing companies during the last decade, enzymes still make up at least 15% of ethanol production costs [1,2]. Reduction of saccharification costs is thus an important prerequisite for commercialization of biomass saccharification and second generation ethanol production processes. Various approaches have been suggested to improve the enzyme economy including higher specific activity of individual enzymes, improved thermal stability, additional enzyme components and better synergistic operation, reduction of end-product inhibitions, more efficient production systems, including consolidated processes, as well as recycling of enzymes [3-6].

Due to the complex structure of lignocellulosic biomass, the action of cellulolytic enzymes including cellobiohydrolases (CBHs), endoglucanases (EGs), lytic polysaccharide monooxygenases (LPMOs) and β-glucosidases (BGs) and various hemicellulases, especially xylanases (XYLs) are required for efficient saccharification of lignocellulosic biomass [7]. Two types of CBHs hydrolyze cellulose simultaneously from the reducing (CBH I, EC 3.2.1.176) and non-reducing ends (CBH II, EC 3.2.1.91), while EGs (and LPMOs) introduce new chain ends for CBHs. *Tr*Cel7A (CBH I) is the major enzyme secreted by the well-studied mesophilic fungus *Trichoderma reesei*, forming approximately 80% of total secreted proteins.

In general, glycoside hydrolases (GHs) degrading insoluble polysaccharides have a bidomain structure with a carbohydrate-binding module (CBM) attached to the catalytic core domain by a flexible, glycosylated linker [8]. CBMs are classified into CBM families based on their sequence similarity and predicted structure-function relationships, and cellulose-binding affinity has been reported for members of 19 CBM families out of 56 [9]. The main proposed role of CBMs is to increase the effective enzyme concentration on the polysaccharide surface, thus targeting the catalytic module to the substrate [10,11]. Experimental challenges are caused by the nonlinear kinetics and multiple binding modes of cellulases. Catalytic core domains lacking CBM have been shown to bind on cellulose with reduced affinity compared to their native proteins at low (1 to 10 g/l) substrate concentrations [12-14]. The correlation between the amount of bound enzyme and activity is, however, not clear. The CBMs have not been shown to increase the catalytic activity towards cellulose, and CBHs with and without CBM proceed along the cellulose chain with a similar speed [15,16]. Remarkably, the CBM has been even observed to decrease the specific activity of the adsorbed enzyme by presumably preventing the processive hydrolysis of cellulose through unproductive binding [17]. It has been suggested that the rate of cellulose hydrolysis is governed by the dissociation rate constant (koff), which is low for processive CBHs [16].

We have recently shown that the CBMs are not needed for efficient hydrolysis if the concentration of the solid substrate is increased at least to about 10% dry matter (DM) [18]. Thus, the presence of CBM can be counterbalanced by reducing the amount of water in the hydrolytic system, which increases the concentration of enzymes in the proximity of the substrate and enhances the probability of adsorption and subsequent catalysis of the core enzymes on the substrate [18]. The same effect was observed with cellulases which naturally occur with (*T. reesei*) or without (*Thermoascus aurantiacus*) a CBM [19]. At elevated substrate concentrations, the thermostable, naturally CBM-lacking *T. aurantiacus* cellulases reached about the same hydrolysis yields as the corresponding enzymes linked to family 1 CBMs [19].

A feasible industrial process for production of bioethanol from lignocellulosic biomasses requires high DM content throughout the process. The DM content of the biomass feed to pretreatment can be as high as 40% and the DM content during hydrolysis approximately 25% [20]. The process configuration, for example the severity of pretreatment, enzyme loading, and residence time in hydrolysis and fermentation, can be adapted to match the performance of the enzymes and thereby optimize the utilization of the enzymes [21]. One potential way to reduce the costs of the enzymatic hydrolysis would be to recycle the enzymes. The advantage of the CBM-lacking enzymes is that a significantly higher share can be recovered non-bound and active after the hydrolysis [18], as compared to the CBM-containing enzymes, of which a higher share remains bound especially to lignin containing substrates. During recycling, loss of enzymes occurs due to unproductive binding and denaturation of enzymes [22,23].

Thermostable CBHs from several fungi have been recently characterized [24,25], and the cellulases of *T. aurantiacus*, naturally secreted without CBM, were found to act efficiently especially on natural cellulosic substrates [19]. The aim of the present work was to further explore these thermostable, CBM-lacking enzymes in order to reach high conversion yields in industrial conditions with high DM consistency. The aim was also to compare the action of cellulases with and without CBM and to evaluate the recyclability potential of the CBM-lacking enzymes.

## Results and discussion

### Comparison of enzymes with and without CBMs

Previously, we have shown that the cellulases *Ta*Cel7A and *Ta*Cel5A of *T. aurantiacus*, naturally lacking the carbohydrate-binding domains, were able to hydrolyze pretreated wheat straw to the same extent as the corresponding enzyme constructs provided with CBMs (*Ta*Cel7A + *Tr*CBM and *Ta*Cel5A + *Ct*CBM) [19]. In our previous experiments, using substrate concentrations above 10% DM the degree of hydrolysis remained fairly low, below 50% of theoretical, mainly due to the short hydrolysis time (24 hours). Therefore, laboratory-scale experiments were carried out to reach a higher conversion yield by increasing enzyme dosage and prolonging hydrolysis time.

**Figure 1:**
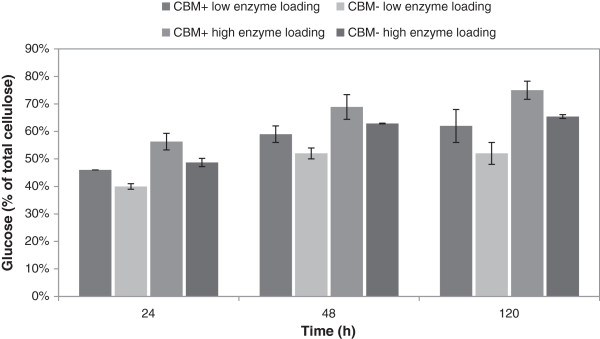
**Glucose yields of pretreated wheat straw with various loadings of *Thermoascus aurantiacus *enzyme preparations.** Glucose yields in small-scale hydrolysis experiments of pretreated wheat straw (consistency 20% DM) by enzyme preparations containing CBH I with (CBM+) or without (CBM-), after 24, 48 and 120 hours of hydrolysis, temperature 50°C. Enzyme loadings are shown in Table 1. CBH, cellobiohydrolase; CBM, carbohydrate-binding module; DM, dry matter.

Conversion of pretreated wheat straw was studied with thermostable enzymes during a prolonged hydrolysis (up to 120 hours) at high, 20% DM, consistency to reach a high yield for tests at a larger scale. The enzyme mixtures were composed of equimolar amounts of CBH I and EG II (*Ta*Cel7A and *Ta*Cel5A) with or without the CBM to allow direct comparison of enzymes with and without CBM, supplemented with BG and XYL, and used at two enzyme dosages: low CBM-containing 14.3 mg/g DM, low CBM-lacking 12.7 mg/g DM, high CBM-containing 28.7 mg/g DM and high CBM-lacking 25.5 mg/g DM (Table 1). The yields reached especially with the higher dosages were rather high (Figure 1), and the yield obtained with the CBM-containing enzymes within 120 hours was above 70%. Using the lower dosages, high hydrolysis yields were not reached even in a prolonged hydrolysis of 120 hours. Taking into account the high DM consistency and that only CBH I (Cel7A) and EG II (Cel5A) were used, in addition to XYL and BG, the yields can be considered satisfactory. The yields reached with the CBM-lacking enzymes were, however, constantly about 10% lower than with enzymes containing the CBM, and the hydrolysis rate seemed to be somewhat slower than with CBM-containing enzymes. Although the liquefaction and solubilization of the substrate may decrease the probability of the enzyme to bind with the substrate, a surprisingly high yield, above 60% glucose of total, could be obtained even with the mixture of enzymes lacking the CBM after 120 hours.

**Table 1:** Enzyme mixtures and their loading in the small scale experiments

**Enzymes**	**Components in enzyme mixtures**		**Dosage of enzymes molar basis: μmol/g DM**		**Dosage of enzymes weight basis: mg/g DM**	
	**CBM+**	**CBM-**	**Low**	**High**	**Low**	**High**
*Ta*Cel7A		+	0.20	0.40	9.4	18.8
*Ta*Cel7A + *Tr*CBM	+		0.20	0.40	10.6	21.2
*Ta*Cel5A		+	0.05	0.10	1.7	3.4
*Ta*Cel5A + *Ct*CBM	+		0.05	0.10	2.1	4.2
*Ta*Xyn10A	+	+	0.05	0.10	1.6	3.3
*Ta*Cel3A	+	+	0.01	0.02	1,000.0^a^	2,000.0^a^

Low and high dosages of enzyme proteins (as μmol or mg/g DM substrate) used in laboratory-scale hydrolysis experiments. Enzyme mixtures showing the components in CBM+ and CBM- enzyme mixtures. The total protein loading of the used enzymes were on the low level, 14.3 mg/g (CBM+) and 12.7 mg/g (CBM-), and on the high enzyme level, 28.7 mg/g (CBM+) and 25.5 mg/g (CBM-). ^a^BG dosed as nkat/g DM. BG, β-glucosidase; CBM, carbohydrate-binding module; DM, dry matter.

As compared with results obtained with the lower protein amount after the shortest, 24 hours of hydrolysis time, glucose yields were clearly higher than those previously obtained with the same enzymes [19] at high consistency. An inversely proportional relationship has often been shown between the DM content in hydrolysis or simultaneous saccharification and fermentation (SSF) experiments and cellulose conversion [26]. In high consistency hydrolysis, the sugar yields have been fairly low, due to problems with mass transfer and insufficient mixing [27], inhibition of enzymes by end-products [28] or by-products formed during pretreatment [29].

**Figure 2:**
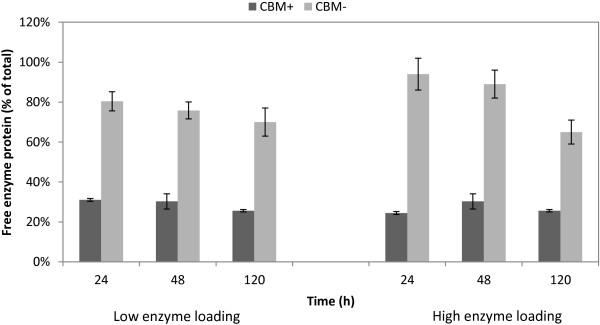
**Free proteins in small-scale hydrolysis of pretreated wheat straw.** Two dosages of *Thermoascus aurantiacus* enzymes with and without the CBMs were used. Samples were analyzed after 24, 48 and 120 hours of hydrolysis. The amounts of free enzymes are expressed as the percentage of total original loaded proteins. Dark grey, CBM-containing (CBM+); light grey, CBM-lacking (CBM-). CBM, carbohydrate-binding module.

The major benefit of using enzymes without CBM would be their better recyclability; *that is,* the higher amount of free enzymes after the hydrolysis. Thus, as observed previously [19] a significantly higher portion of the enzymes without CBM remained free after the hydrolysis (Figure 2). About 25 to 35% of the enzymes with CBM could be recovered after the hydrolysis, whereas about 70 to 80% of the cellulases without the CBM were free in the solution. The recovery of the traditional proteins with CBM after hydrolysis was in accordance with previously reported results from experiments with pretreated biomass containing lignin [30-32].

### Partial replacement of *Ta*Cel7A by *Ct*Cel6A

**Figure 3:**
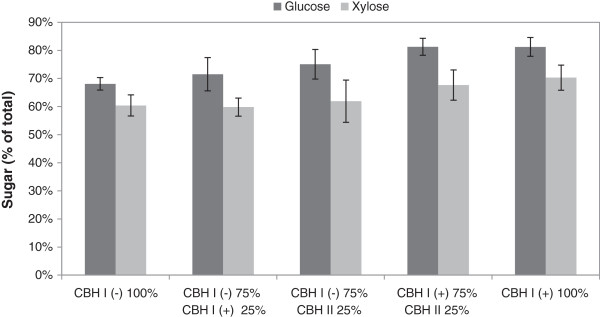
**Glucose and xylose yields in small-scale hydrolysis experiments of pretreated wheat straw.** Combinations of CBHs: 25% of native CBH I (CBM-) (*Ta*Cel7A) was replaced by the CBM containing CBH I (CBH+) (*Ta*Cel7A + *Tr*CBM) or by the CBM containing CBH II from *Chaetomium thermophilum* (*Ct*Cel6A). The enzyme preparations also contained EG II (CBM-) (*Ta*Cel5A), XYL and BG from *Thermoascus aurantiacus.* Substrate consistency was 20% DM, hydrolysis time 48 hours and temperature 50°C. BG, β-glucosidase; CBH, cellobiohydrolase; CBM, carbohydrate-binding module; DM, dry matter; EG, endoglucanase; XYL, xylanase.

The enzyme preparation used in the experiments contained only two of the major cellulases, Cel7A (CBH I) and Cel5A (EG II), both naturally lacking the CBM. In an attempt to increase the hydrolysis yields, the addition of CBHs with CBMs was studied. Thus, 25% of the CBH I without the CBM was replaced by the CBM containing CBH I from *T. aurantiacus* or by the naturally CBM containing CBH II from *Chaetomium thermophilum* (Figure 3). The amount of EG II was kept constant as well as the molar ratio of the CBHs and EG. Replacing 25% of *Ta*Cel7A, CBM-lacking CBH I, by the CBM containing *Ta*Cel7A + *Tr*CBM increased the yield of glucose slightly, whereas replacement of the same by *Ct*Cel6A, a naturally CBM containing CBH II, increased the yield slightly more, by about 11% compared with the CBM-lacking enzyme alone. This may reflect the relatively more important role of a CBM in the less processive CBH II. On the other hand, somewhat surprisingly, replacement of 25% of the CBH I by the CBH II, both with a CBM, had no major effect on the hydrolysis yield in the conditions used (Figure 3). The importance of CBH II (family 6A) in hydrolysis has been shown to depend on the raw material used, as well as on the overall composition of the preparation used, as reviewed previously [33]. As expected, formation of xylose in the hydrolysis seemed to correlate with the overall increase of the hydrolysis yield (Figure 3).

### Enzyme mixtures and hydrolysis conditions in small-scale experiments

In order to optimize the conditions to be used in the industrial high consistency (25%) system, hydrolysis experiments were carried out varying the amount of enzymes with or without the CBM and by omitting the heat treatment (Table 2). In previous experiments, the enzyme preparations were heat treated in order to remove the minor side activities present in the preparations. In the production strains designed for the expression of the individual thermostable enzymes, the major cellulase genes had been deleted. The background proteins produced by *T. reesei* contained minor EGs, xylanolytic enzymes and other side activities, eventually improving the hydrolysis yields. The hydrolysis temperature was 50°C, where most of these minor activities were detectable at least for a short period. In order to retain these positive activities in the larger-scale experiments, enzyme preparations without heat treatment were studied. The results showed that at a high enzyme dosage of 28.8 mg/g DM, the glucose yield reached 93% and 82% of the theoretical yields by enzyme mixtures with and without the CBMs, respectively (Table 2). Addition of polyethylene glycol (PEG) 6000 had a further positive effect on the hydrolysis by both enzyme mixtures, previously known to improve the hydrolysis [34]. For the large-scale experiments, a protein dosage of 17.3 mg/g DM was chosen in order to reach a desirable hydrolysis yield with a reasonable protein loading. In large-scale experiments PEG 6000 was also included.

**Table 2:** Sugar yields in small-scale hydrolysis experiments of pretreated wheat straw using various protein loadings

**Total loaded protein (mg/g DM)**	**Hydrolysis yield, % of total carbohydrates**	
	**CBM-containing**	**CBM-lacking**
7.2	39.4 (6.5)	37.9 (3.6)
14.4	60.5 (5.4)	60.0 (0.8)
17.3	84.1 (1.5)	78.5 (0.4)
28.8	93.3 (0.5)	81.9 (1.3)
28.8^a^	94.4 (1.6)	90.7 (1.9)
43.2	88.6 (4.6)	86.6 (2.2)

Sugar yields in small-scale hydrolysis experiments of pretreated wheat straw (consistency 25%) using various protein loadings by enzyme mixtures containing CBH I with (CBM+) or without (CBM-), EG II, XYL and BG of *Thermoascus aurantiacus.* The ratio of enzymes (protein-based) was CBH I:EG II:XYL 6:1:1. BG was dosed as nkat/g dw following the ratios of other proteins. ^a^Addition of PEG, 1% of substrate DM. Hydrolysis 72 hours, temperature 50°C. Hydrolysis yields are expressed as the percentage of total carbohydrates, measured as reducing sugars. Standard deviations are in parentheses. BG, β-glucosidase; CBH, cellobiohydrolase; CBM, carbohydrate-binding module; DM, dry matter; EG, endoglucanase; PEG, polyethylene glycol; XYL, xylanase.

### Hydrolysis and fermentation in industrial conditions

**Figure 4:**
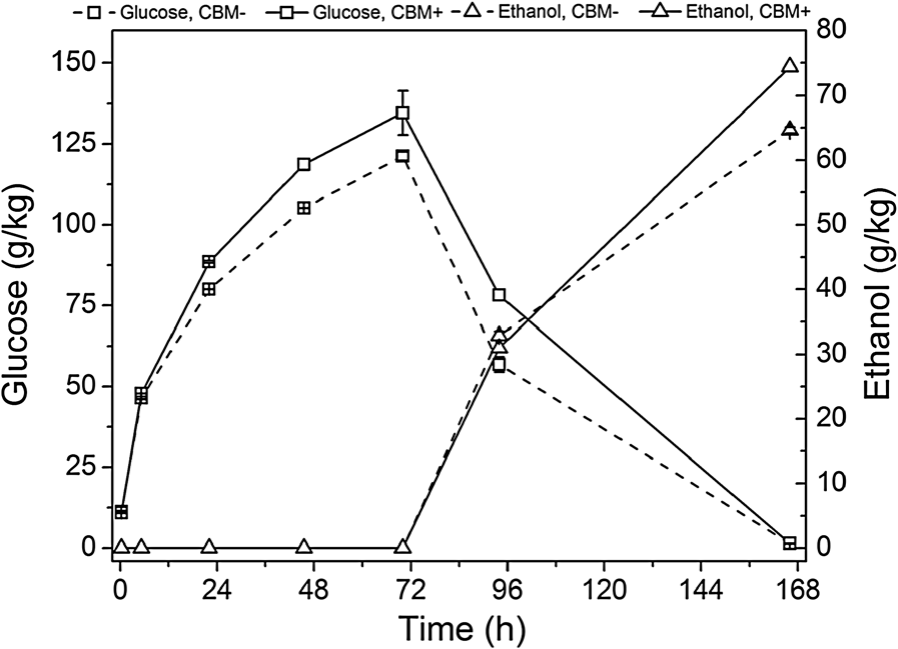
**Formation of glucose and ethanol during the hydrolysis and fermentation in large-scale experiments.** Enzyme dosage was 17.3 mg/g DM, hydrolysis temperature 50°C, inoculation of yeast at 70 hours, fermentation temperature 33°C. DM, dry matter.

**Figure 5:**
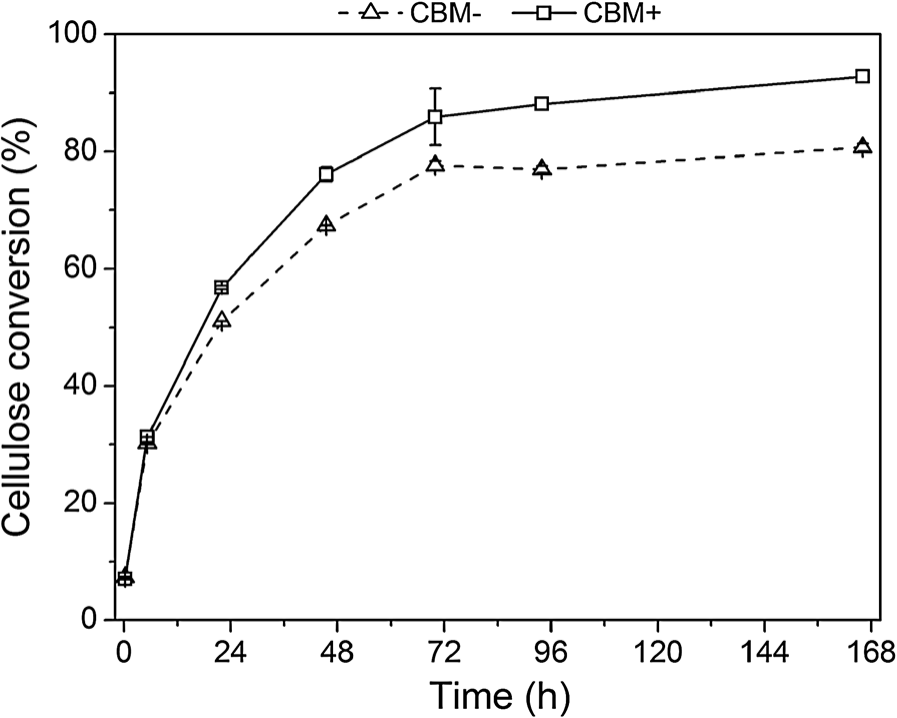
**Conversion yield of pretreated straw after combined hydrolysis and fermentation in large-scale experiments.** Conversion yield of pretreated straw after 70 hours of hydrolysis and 96 hours of fermentation in large-scale experiments. The yield was calculated from Equation 1.

In order to mimic the industrial hydrolysis and fermentation conditions, large-scale experiments were carried out at the DONG Energy’s Inbicon pilot plant (Skærbæk, Denmark). Although fairly efficient mixing could be achieved even in the small-scale experiments, results in pilot-scale provide a more reliable comparative basis. The enzymes available for these large-scale experiments included the *T. aurantiacus* CBH I, EG II, XYL and BG, at a total dosage of 17.3 mg/g DM. The process design consisted of a hydrolysis stage extending for 70 hours at 50°C, followed by a fermentation stage of 96 hours. Glucose started to accumulate at a very high rate already at the beginning of the hydrolysis, reaching a high concentration of 135 and 120 g/l using enzymes with and without the CBM, respectively, in 70 hours (Figure 4). After this stage, the temperature was decreased to 33°C and the dry yeast was added. The fermentation started vigorously and the concentration of glucose declined rapidly. Glucose was completely consumed at the last measurement point (168 hours). Based on the glucose consumption rate, however, it can be expected that glucose was already consumed between the last two measurement points of 94 and 168 hours. The final ethanol concentration reached approximately 75 and 65 g/l using enzymes with and without the CBM, respectively (Figure 4). The total conversion yields, as calculated from Equation 1, were 92% and 80% of the theoretical with enzymes containing or lacking the CBM, respectively (Figure 5). A low amount of lactic acid was formed during the fermentations (data not shown). In the hydrolysis with the CBM-containing enzymes, the concentration of xylose was 9.1 g/l and with the CBM-lacking enzymes 8.3 g/l, corresponding to the hydrolysis yields of cellulose; *that is,* formation of glucose by these enzymes. This may be due to decreased hydrolysis of xylans with mixtures of enzymes lacking the CBM, especially by *Ta*Cel5A, or by the close physical proximity of xylans and cellulose in the substrate.

### Residual activities of the CBHs

**Figure 6:**
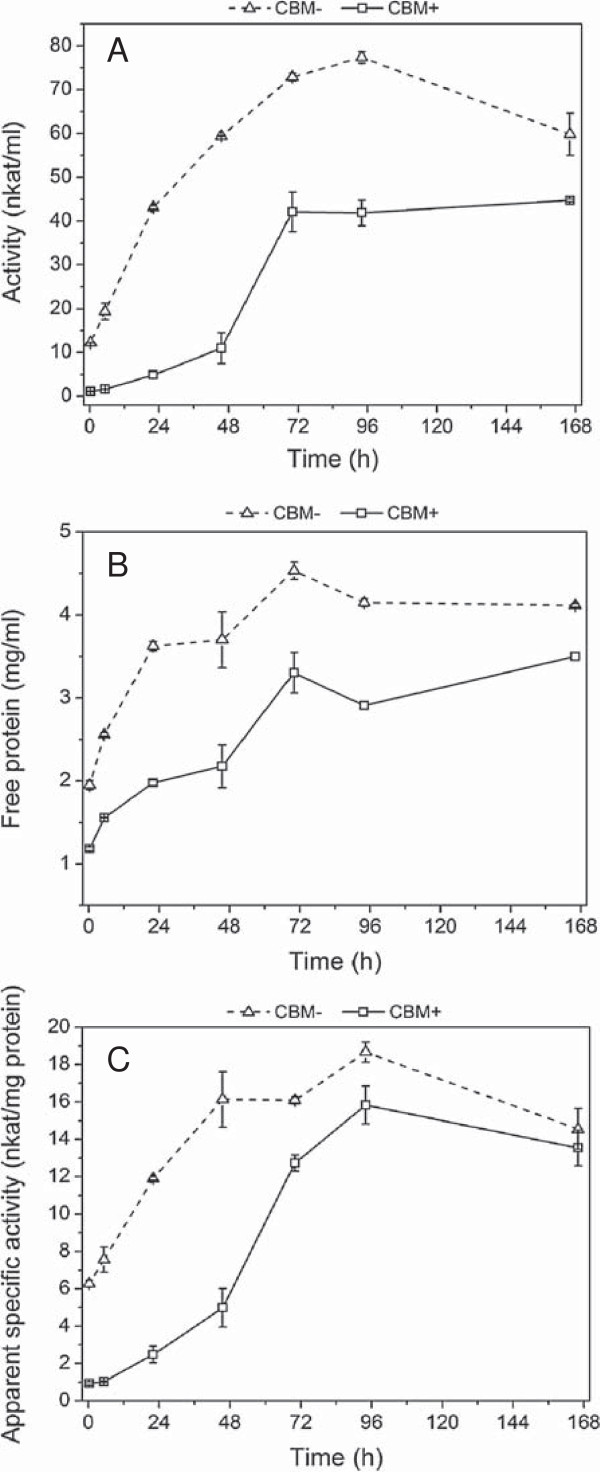
**Analysis of enzyme activity and protein during the hydrolysis and fermentation of pretreated wheat straw. (A)** MUL activity, **(B)** free protein and **(C)** the ratio of activity and protein in large-scale experiments. MUL, 4-methylumbelliferyl-β-D-lactoside.

The concentration and activities of the free enzymes with and without CBM during the larger scale hydrolysis and fermentation experiments (at the Inbicon pilot unit) were also measured. Initially, after mixing the enzymes and substrate, about 55% of enzymes lacking the CBM and 73% of enzymes with CBM were bound to the substrate (Figure 6B). The 4-methylumbelliferyl-β-D-lactoside (MUL) activity (Figure 6A) and amount of free proteins (Figure 6B) of the CBM-lacking enzymes during the hydrolysis and fermentation were clearly higher throughout the experiments as compared with enzymes containing the CBM. As observed earlier [18], the CBM-lacking enzymes were significantly less bound to the substrate than the enzymes with CBM during the most active phase (6 to 48 hours) of the hydrolysis, as indicated by the higher free protein concentration and enzyme activity. The difference in substrate binding of enzymes, however, affected surprisingly little the rate of sugar formation during the early stage of the hydrolysis (Figure 4). During the initial phase of the hydrolysis, the ratio of the MUL activity to the total free protein (‘apparent specific MUL activity’) in experiments with the CBH-lacking enzymes was up to four times higher as compared to enzymes with the CBM (Figure 6C).

The results clearly show that the CBM-lacking enzymes, in spite of being less bound to the substrate throughout the hydrolysis, were able to produce almost the same level of hydrolysis. During the course of hydrolysis, both the MUL activity (Figure 6A) and amount of free proteins (Figure 6B) detected in the supernatant increased continuously due to depletion of available substrate sites as a result of solubilization of the solid substrate, as observed previously [35]. Thus these results support the previous postulation regarding similar yield from CBM-containing enzymes and CBM-lacking enzymes during hydrolysis at high consistency. Despite having lower adsorption rate in low consistency [15], higher substrate concentration enables increased probability for binding and ensuing processive catalysis for CBM-lacking enzymes [18]. Coupled with faster desorption rate of CBM-lacking enzymes [14], which is rate-limiting for processive cellulases due to the presence of obstacles in the substrate [16], this conceivably makes the catalysis almost equally fast (Figure 4). Nevertheless, the exact mechanism of the CBM-lacking enzymes is still unknown and subject to further studies. Several factors seem to affect the efficiency of the action of CBM-lacking enzymes under high substrate consistency, including the need of CBM in the individual cellulases throughout the hydrolysis process and their synergistic mechanisms.

After 70 hours of hydrolysis, the temperature was decreased and yeast was inoculated together with addition of yeast extract; the total added amount corresponding to 1 g/l. The addition is clearly seen as a peak in the amount of free proteins (Figure 6B). The added proteins were slowly consumed by the yeast with simultaneous changes in the concentrations of free enzymes in the fermentation broth. After prolonged fermentation time, the MUL activity of the CBM-lacking enzymes was clearly decreased, leading also to a decrease in the approximated specific activity (Figure 6C). In contrast, the CBM-containing enzymes retained their activity relatively better in the solution, although being lower throughout the hydrolysis and fermentation. It is obvious that enzymes lacking the CBM seemed to be more prone to inactivation than the counterparts containing CBM. Expectedly, the proteins were inactivated or denaturated by the high concentration of ethanol, leading to decreased MUL activity (and eventually decreased concentration of soluble protein) of the CBM-lacking enzymes. Ethanol is known to inhibit the cellulases of *T. reesei* progressively up to an ethanol concentration of about 7% [36], and ethanol concentrations of 2 to 9% (v/v) have been observed to inhibit the hydrolysis of crystalline cellulose [37]. It has been found that hydrophobic interaction of ethanol with cellulases could alter their structure resulting in denaturation and subsequent degradation of CBHs. This denaturing effect of ethanol can also be accelerated with high temperature and extreme pH [38].

The fermentation time was originally long to ensure complete hydrolysis and fermentation, although it could have been shorter to recover the more sensitive CBM-lacking enzymes. To date, no systematic study has been reported on the effect of CBM on the stability of cellulases in the presence of ethanol, although the CBM of *Tr*Cel7A is known to stabilize the enzyme thermally, increasing the overall melting point of the enzyme [39]. Therefore, the high ethanol concentration at the end of the fermentation may be one of the factors decreasing the amount and activity of the CBM-lacking enzymes. More experiments should be carried out to reveal the underlying inhibitory factors.

### Recyclability potential of the CBM-lacking enzymes

Preliminary experiments on the recyclability potential of the CBM-lacking enzymes were carried out using enzyme solutions concentrated after the primary hydrolysis experiments in small and large scales. The ultrafiltration step after the large-scale experiments also removed the sugars and ethanol from the enzyme solutions, which may inhibit hydrolysis. The recovered enzymes were tested and compared with fresh enzymes in small-scale experiments. The recycled and fresh enzymes were applied at the same protein dosages (10 mg/g) in the recyclability tests. Enzymes recycled from small laboratory-scale experiments using heat treated enzyme preparations (without fermentation) produced almost equal sugar yields, 93% of the yield with the fresh enzymes: the recycled CBM-lacking mixture yielded 38.9% of total carbohydrates as compared to 41.9% obtained with the fresh CBM-lacking enzyme mixture (Table 3). The hydrolysis yields obtained with the CBM-lacking enzymes from the large-scale experiment (30.9% of total carbohydrates) were, however, clearly lower than with the fresh enzymes (52.1% of total carbohydrates). By replacing 30% of the recycled CBM-lacking enzymes the hydrolysis yield reached 45.9% of total carbohydrates, 88% of the reference. The recovered activity of enzymes without the CBM was generally higher than in previously reported experiments carried out with commercial, CBM-containing enzymes at industrial conditions. Lindedam *et al*. [6] reported that at low DM (12%) 12 to 68% of the total cellulolytic activity could be recovered in the fermentation broth depending on the cellulase preparation and the process conditions used. The results clearly show the recycling potential of the CBM-lacking enzymes, although low enzyme protein amounts were used in the first recycling experiments. Presumably due to the long exposure of the enzymes to high temperature and to ethanol in the larger-scale experiments, the yields were lower than expected, which could be prevented by avoiding the denaturation or inhibition of the enzymes. Clearly, the recycling conditions should also be optimized with respect to the recovery methods, as well as optimal enzyme mixtures (with or without CBM) designed for the hydrolysis and eventual addition of ‘make-up’ enzymes.

**Table 3:** Hydrolysis of pretreated wheat straw with recycled enzymes

**Enzymes in recycling experiments**	**Hydrolysis yield, % of total carbohydrates**
**Small-scale**	
Reference (fresh CBM-lacking enzymes)	41.9 (1.2)
Recycled 100% (CBM-lacking enzymes)	38.9 (0.7)
**Large-scale**	
Reference CBM-containing (100%)	53.6 (4.0)
Reference CBM-lacking (100%)	52.1 (0.4)
Recycled CBM-lacking (100%)	30.9 (1.1)
Recycled CBM-lacking (70% + fresh 30%)	45.9 (4.3)

Hydrolysis yields by recycled enzymes in small- (20% DM) and large-scale (25% DM) hydrolysis experiments of pretreated wheat straw for 72 hours at 50°C. In small-scale experiments the *Thermoascus aurantiacus* enzymes CBH I (CBM-), EG II, XYL and BG were collected after first hydrolysis (72 hours at 50°C), concentrated and dosed on the same protein load (10 mg/g DM) as the reference enzyme mixture. In large-scale experiments the enzymes were collected after fermentation and concentrated for reuse. Protein dosage in all experiments was 10 mg/g DM. Enzymes used in small-scale experiments were heat treated prior to first hydrolysis, while not in the large-scale experiments. The ratio of enzymes (protein-based) for the first hydrolyses was CBH I:EG II:XYL:BG 15:3:2:1. Hydrolysis yields are expressed as the percentage of total carbohydrates, measured as reducing sugars and the standard deviations are in parentheses. BG, β-glucosidase; CBH, cellobiohydrolase; CBM, carbohydrate-binding module; DM, dry matter; EG, endoglucanase; XYL, xylanase.

The high consistency processing conditions resemble the natural, fairly dry environment prevailing during fungal degradation of lignocellulosic substrates, such as wood, litter and other plant cell walls. Recently, several fungi have been found to produce cellulases lacking CBM [18]. The need for cellulose-binding domains in cellulases may depend on the growth habitat of various organisms growing on lignocellulosic substrates. Thus, the degradation processes of cellulose by anaerobic bacteria in aqueous environments varies significantly to those of fungi growing, *for example,* on decaying wood in forests. Results of the present work may reflect these differences and thus increase our understanding of the role of water in microbial environments and its possible consequences on the evolution of different cellulolytic systems in various microorganisms.

## Conclusions

In this article, we showed that enzyme mixtures lacking the CBM could be successfully used in larger scale at high DM consistency using efficient mixing, resulting in cellulose conversion yields above 80% of theoretical. Based on preliminary experiments, enzyme mixture composed of enzymes without CBM could also be efficiently recycled. The activity and amount of recoverable enzymes after the hydrolysis was significantly higher using enzymes without the binding domains. The hydrolysis yields obtained with the two cellulases lacking CBM (*Ta*Cel7A and *Ta*Cel5) could be improved by prolonging the hydrolysis time and by partially replacing the CBH GH family 7 (GH7) with GH family 6 (GH6). The action mechanisms of the CBM-lacking, mostly non-adsorbed enzymes should be further studied to gain deeper understanding of the binding/desorption mechanisms of cellulases, and to design optimal hydrolysis and recycling processes.

## Methods

## Materials

Wheat straw was pretreated at the Inbicon pilot plant at 195°C for 15 minutes (with a severity of 3.96) using a hydrothermal steam pretreatment without addition of chemicals [21]. The raw material in small-scale experiments was air-dried to DM content of 42.5% to enable the process at higher consistencies. The DM of the straw used in large-scale experiments was 28.3%. Duplicate chambers were fed with samples from different batches. The composition of the substrates was determined after acid hydrolysis [40] by HPLC using an UltiMate 3000 HPLC (Thermo Scientific Dionex, Sunnyvale, CA, USA) equipped with a refractive index detector (Shodex RI-101; Shodex Munich, Germany). The separation was performed on a Rezex-RHM monosaccharide column (Phenomenex, Macclesfield, UK) at 80°C with 5 mM H_2_SO_4_ as eluent at a flow rate of 0.6 ml/min. The composition of the pretreated substrate was determined using Laboratory Analytical Procedures established by the National Renewable Energy Laboratory [40]. All chemicals used were of analytical grade and purchased from Sigma-Aldrich (St Louis, MA, USA) or Merck (Whitehouse Station, NJ, USA). The pretreated wheat straw contained 51.1 ± 1.2% of cellulose, 4.0 ± 0.0% of xylan and 34.1 ± 0.4% of lignin on dry weight basis (averages of three different samples analyzed in triplicate).

### Enzymes

For hydrolysis experiments, thermostable enzymes with and without CBM from *T. aurantiacus* were used: the intact CBH I without CBM (*Ta*Cel7A) and fused with *T. reesei* CBM (*Ta*Cel7A + *Tr*CBM), and the native EG II without CBM (*Ta*Cel5A) and fused to the linker and CBM of *C. thermophilum* Cel7A (*Ta*Cel5A + *Ct*CBM). In addition, XYL *Ta*Xyn10A and BG *Ta*Cel3A from *T. aurantiacus* were added to all experiments. The CBH II from *C. thermophilum* (*Ct*Cel6A), naturally carrying a CBM, was added to replace part of the *T. aurantiacus* CBH I in some experiments.

All thermostable enzymes, including the CBH II from *C. thermophilum* (*Ct*Cel6A) were expressed in *T. reesei* as described previously [19]. The expression cassettes were transformed into *T. reesei* industrial production strains that lack the genes *cbh1*, *cbh2*, *egl1* and *egl2*, encoding for CBH I (*Tr*Cel7A), CBH II (*Tr*Cel6A), EG I (*Tr*Cel7B) and EG II (*Tr*Cel5A), respectively. The XYL *Ta*Xyn10A and BG *Ta*Cel3A were produced accordingly [19]. All thermostable enzyme preparations were adjusted to pH 6.0 and treated at 60°C for 2 hours to inactivate the background *T. reesei* enzymes. For the large-scale experiments in industrial conditions the preparations were used as such.

The activity of CBH I was measured using the soluble MUL (Sigma-Aldrich) substrate with 4-methylumbelliferone (Sigma-Aldrich) standard as described previously [41]. Analyses were done in duplicates. The hydrolysates were first diluted twofold with 0.05 M Na-citrate buffer, pH 5, centrifuged, after which the activity was determined.

### Small-scale enzymatic hydrolysis

The substrate was hydrolyzed with two dosages (low and high) of enzymes (CBH I *Ta*Cel7A, EG II *Ta*Cel5A, XYL TaXyn10A and BG *Ta*Cel3A) using equimolar amounts of cellulases with or without CBM, using a ratio of *Ta*Cel7A to *Ta*Cel5A of 4:1 (Table 1). At low and high dosage levels, a total of 0.25 or 0.50 μmol/g DM of *Ta*Cel7A and *Ta*Cel5A with (CBM+) or without CBM (CBM-) were used. The used low dosages of the *Ta*Cel7A (CBM+) and *Ta*Cel7A (CBM-) were 10.6 and 9.4 mg protein/g DM, and of *Ta*Cel5A (CBM+) and *Ta*Cel5A (CBM-) were 2.1 and 1.7 mg protein/g DM, respectively. The used high dosages of *Ta*Cel7A (CBM+) and *Ta*Cel7A (CBM-) were 21.2 and 18.8, and those of *Ta*Cel5A (CBM+) and *Ta*Cel5A (CBM-) were 4.2 and 3.4 mg protein/g DM, respectively. To compare the role of CBHs, *Ta*Cel7A (CBM-) was either partially replaced (25% on molar basis) with *Ta*Cel7A (CBM+) or with *Ct*Cel6A (naturally containing a CBM). For comparison, *Ta*Cel7A (CBM-) was also totally replaced by *Ta*Cel7A (CBM+). In these experiments, the higher dosage level was used.

The laboratory-scale hydrolysis experiments were carried out in triplicates at 20% or 25% DM (w/w) consistency in 0.05 M Na-citrate buffer, pH 5, in a volume of 2 ml (10-ml tubes) at 50°C. The samples were mixed with combined gravity and vortex mixing for 24 to 120 hours using an Intelli-Mixer RM-2 (ELMI, Riga, Latvia) with u2 mode at 35 rpm, exerting continuous variable intensity small amplitude vortexing with second speed counter-clockwise rotation [19]. PEG with an average molecular weight of 6,000 g/mol (PEG 6000) was added before addition of enzyme at a loading of 10 g/kg DM.

After hydrolysis in small-scale, samples withdrawn from experiments on different DM concentrations were cooled and diluted with buffer to a final 5% DM substrate concentration. The samples were centrifuged (15 minutes, 2,500 rpm) to separate the solid and liquid phases. A part of the supernatants was frozen for measurements of protein, and the remaining supernatants were boiled for 10 minutes for determination of glucose. The hydrolysis yields were calculated as the percentage of the theoretical maximum conversion of total glucose (or carbohydrates) in the substrates.

### Large-scale enzymatic hydrolysis and ethanol fermentation

Hydrolysis and fermentation experiments at pilot-scale using high DM consistency were carried out at the Inbicon pilot plant of DONG Energy in a specially designed six-chamber reactor with a minimum working volume of 10 kg employing the principle of free-fall mixing as described earlier [42]. The DM content of pretreated wheat straw was adjusted to 25% by addition of water and the pH was adjusted to 5 with Na_2_CO_3_. PEG with an average molecular weight of 6,000 g/mol (PEG 6000, 10 g/kg DM) was added before addition of enzyme. The cellulases *Ta*Cel7A and *Ta*Cel5A (with or without CBM), as well as XYL (*Ta*Xyn10A) and BG (*Ta*Cel3A) were dosed on the basis of protein at a ratio of 15:3:2.5:1, respectively. The total amount of loaded protein was 17.2 mg/g DM. In large-scale experiments the enzymes were not heat treated. The biomass was hydrolyzed for 70 hours at 50°C before it was cooled to 33°C, then the yeast Thermosacc Dry (2 g/kg DM; Lallemand Ethanol Technology, Milwaukee, WI, USA) and yeast extract (4 g/kg DM; Merck) were added afterwards and the sample was collected at the start of the fermentation. The fermentation was continued for 96 hours. The experiments with both types of enzymes, CBM-containing and CBM-lacking, were carried out in duplicates. Samples withdrawn during the experiments were not diluted prior to separation of solids.

### Recycling of enzymes

Enzyme recycling experiments were carried out at the laboratory-scale using proteins collected from small- and large-scale experiments. At small-scale, samples were collected after 72 hours of hydrolysis at 50°C and in large-scale after hydrolysis (70 hours, 50°C) and extended fermentation (96 hours, 33°C). Supernatants containing the CBM-lacking enzymes from both experiments were concentrated by 10 kDa Amicon membranes by centrifuging at 4°C and 3,000 rpm (Merck). The amount of proteins in each filtrate was determined and dosed (10 mg/g DM) at the same level for the recycling tests. Recycled and fresh enzyme mixtures were compared in hydrolysis experiments, carried out as described above (72 hours, 50°C). PEG was not added to the recycling experiments.

### Analysis of hydrolysis and fermentation products

The amount of reducing sugars was determined using the dinitrosalicylic acid (DNS) method with glucose as standard [43]. Monosaccharides in the small-scale hydrolysates were analyzed using high performance anion exchange chromatography with pulsed amperometric detection (HPAEC-PAD) equipped with a 2707 autosampler (Waters Corporation, Milford, MA, USA), 515 HPLC pumps (Waters) and a 2465 pulsed amperometric detector (Waters), provided with Empower 2 software for instrument control and data analysis [44]. In the large-scale experiments, sugars, organic acids and ethanol were quantified by HPLC using an UltiMate 3000 HPLC (Thermo Scientific Dionex) equipped with a refractive index detector (Shodex RI-101) as described previously [42,45]. The separation was performed on a Rezex-RHM monosaccharide column (Phenomenex) at 80°C with 5 mM H_2_SO_4_ as eluent at a flow rate of 0.6 ml/min. D-Glucose and D-xylose (Merck) were used as external standards. The conversion of cellulose was calculated from the cellulose content of the pretreated material (*WT*%_*Cel*_), the amount of pretreated biomass (*m*_*biomass*_), the DM content (*DM*%), and the concentrations of cellobiose (*C*_*Cel*_), glucose (*C*_*Glc*_) and ethanol (*C*_*EtOH*_):

Equation 1:

$$\mathrm{Cellulose}\;\mathrm{conversion}=\frac{C_{\mathrm{Cel}}∙\frac{360g/\mathrm{mol}}{342g/\mathrm{mol}}+C_{\mathrm{Glc}}+\frac{C_{\mathrm{EtOH}}}{0.51g\;\mathrm{EtOH}/g\;\mathrm{Glc}}}{m_{\mathrm{biomass}}∙\mathrm{DM\%}∙\mathrm{WT}{\%}_{\mathrm{Cel}}∙\frac{180g/\mathrm{mol}}{160g/\mathrm{mol}}}$$

### Analysis of proteins

Proteins in hydrolysis supernatants were measured by two methods: Lowry and ninhydrin methods [30,46,47]. In laboratory-scale experiments, proteins were precipitated by adding three volumes of acetone (Rathburn Chemicals Ltd, Walkerburn, UK), re-dissolved in Lowry reagent A (20 g/l Na_2_CO_3_ and 4 g/l NaOH) and quantified by the Lowry method using BSA (Sigma-Aldrich) as standard [46]. In large-scale experiments, the protein concentration in supernatant was determined using the ninhydrin method with an alkaline protein hydrolysis as described by Haven and Jørgensen [30]. Supernatants were separated from the solids by centrifugation at 4,200 × *g* for 10 minutes and diluted approximately eight times to obtain protein concentrations below 800 μg/ml and used for the ninhydrin assay. All samples were analyzed in triplicates.

## Abbreviations

BG: β-Glucosidases; BSA: Bovine serum albumin; CBH: Cellobiohydrolase; CBM: Carbohydrate-binding module; DM: Dry matter; DNS: Dinitrosalicylic acid; EG: Endoglucanase; GH: Glycoside hydrolase; HPAEC-PAD: High performance anion exchange chromatography with pulsed amperometric detection; HPLC: High performance liquid chromatography; koff: Dissociation rate constant; LPMO: Lytic polysaccharide monooxygenase; MUL: 4-Methylumbelliferyl-β-D-lactoside; PEG: Polyethylene glycol; SSF: Simultaneous saccharification fermentation; XYL: Xylanase.

## Competing interests

The authors declare that they have no competing interests.

## Authors’ contributions

AP, MØH and DTD performed data collection and analysis, and undertook manuscript writing. TP and LV conceived and designed the study, and critically revised the manuscript. AV conceived and designed the study, and undertook manuscript writing. All authors read and approved the final manuscript.

## References

[B1] Aden A Foust T Technoeconomic analysis of the dilute sulfuric acid and enzymatic hydrolysis process for the conversion of corn stover to ethanol Cellulose 2009 16 535 545 10.1007/s10570-009-9327-8

[B2] Humbird D Davis R Tao L Kinchin C Hsu D Aden A Schoen P Lukas J Olthof B Worley M Sexton D Dudgeon D Process Design and Economics for Biochemical Conversion of Lignocellulosic Biomass to Ethanol NREL Technical *R*eport: NREL/TP-5100-47764 2011 Golden, CO: National Renewable Energy Laboratory

[B3] Zhang YHP Himmel ME Mielenz JR Outlook for cellulase improvement: screening and selection strategies Biotechnol Adv 2006 24 452 481 10.1016/j.biotechadv.2006.03.003 16690241

[B4] Wyman CE What is (and is not) vital to advancing lignocellulosic ethanol Trends Biotechnol 2007 25 4 153 157 10.1016/j.tibtech.2007.02.009 17320227

[B5] Chandel AK Chandrasekhar G Silva MBG da Silva SS The realm of cellulases in biorefinery development Crit Rev Biotechnol 2011 32 3 187 202 21929293 10.3109/07388551.2011.595385

[B6] Lindedam J Haven MØ Chylenski P Jørgensen H Felby C Recycling cellulases for cellulosic ethanol production at industrial relevant conditions: potential and temperature dependency at high solid processes Bioresour Technol 2013 148 180 188 24045205 10.1016/j.biortech.2013.08.130

[B7] Chundawat SPS Beckham GT Himmel ME Dale BE Deconstruction of lignocellulosic biomass to fuels and chemicals Annu Rev Chem Biomol Eng 2011 2 121 145 10.1146/annurev-chembioeng-061010-114205 22432613

[B8] Gilkes NR Henrissat B Kilburn DG Miller RC Warren RAJ Domains in microbial β-1,4-glycanases: sequence conservation, function, and enzyme families Microbiol Rev 1991 55 303 315 1886523 10.1128/mr.55.2.303-315.1991 PMC372816

[B9] Cantarel BL Coutinho PM Rancurel C Bernard T Lombard V Henrissat B The Carbohydrate-Active EnZymes database (CAZy): an expert resource for Glycogenomics Nucleic Acids Res 2009 37 D233 D238 10.1093/nar/gkn663 18838391 PMC2686590

[B10] Reinikainen T Ruohonen L Nevanen T Laaksonen L Kraulis P Jones TA Knowles JKC Teeri TT Investigation of the function of mutated cellulose-binding domains of *Trichoderma reesei* cellobiohydrolase I Proteins 1992 14 4 475 482 10.1002/prot.340140408 1438185

[B11] Boraston AB Bolam DN Gilbert HJ Davies GJ Carbohydrate-binding modules: Fine-tuning polysaccharide recognition Biochem J 2004 382 769 781 10.1042/BJ20040892 15214846 PMC1133952

[B12] van Tilbeurgh H Tomme P Claeyssens M Bhikhabhai R Pettersson G Limited proteolysis of the cellobioydrolase I from *Trichoderma reesei*. Separation of functional domains FEBS Lett 1986 204 223 227 10.1016/0014-5793(86)80816-X

[B13] Tomme P van Tilbeurgh H Pettersson G van Damme J Vandekerckhove J Knowles J Teeri T Claeyssens M Studies of the cellulolytic system of *Trichoderma reesei* QM 9414. Analysis of domain function in two cellobiohydrolases by limited proteolysis Eur J Biochem 1988 170 575 581 10.1111/j.1432-1033.1988.tb13736.x 3338453

[B14] Ståhlberg J Johansson G Pettersson G A new model for enzymatic hydrolysis of cellulose based on the two-domain structure of cellobiohydrolase I Nat Biotechnol 1991 9 286 290 10.1038/nbt0391-286

[B15] Igarashi K Koivula A Wada M Kimura S Penttilä M Samejima M High speed atomic force microscopy visualizes processive movement of *Trichoderma reesei* cellobiohydrolase I on crystalline cellulose J Biol Chem 2009 284 36186 36190 10.1074/jbc.M109.034611 19858200 PMC2794734

[B16] Jalak J Väljamäe P Mechanism of initial rapid rate retardation in cellobiohydrolase catalyzed cellulose hydrolysis Biotechnol Bioeng 2010 106 871 883 10.1002/bit.22779 20506147

[B17] Nakamura A Tsukuda T Auer S Furuta T Wada M Koivula A Igarashi K Samejima M The tryptophan residue at the active site tunnel entrance of *Trichoderma reesei* cellobiohydrolase Cel7A is important for initiation of degradation of crystalline cellulose J Biol Chem 2013 288 19 13503 13510 10.1074/jbc.M113.452623 23532843 PMC3650387

[B18] Várnai A Siika-aho M Viikari L Carbohydrate-binding modules (CBMs) revisited: Reduced amount of water counterbalances the need for CBMs Biotechnol Biofuels 2013 6 30 10.1186/1754-6834-6-30 23442543 PMC3599012

[B19] Le Costaouëc T Pakarinen A Várnai A Puranen T Viikari L The role of carbohydrate binding module (CBM) at high substrate consistency: comparison of *Trichoderma reesei* and *Thermoascus aurantiacus* Cel7A (CBHI) and Cel5A (EGII) Bioresour Technol 2013 143 196 203 23796604 10.1016/j.biortech.2013.05.079

[B20] Larsen J Haven MØ Thirup L Li HW Iversen FK The IBUS process - lignocellulosic bioethanol close to a commercial reality Chem Eng Technol 2008 31 765 772 10.1002/ceat.200800048

[B21] Petersen MØ Larsen J Thomsen MH Optimization of hydrothermal pretreatment of wheat straw for production of bioethanol at low water consumption without addition of chemicals Biomass Bioenergy 2009 33 834 840 10.1016/j.biombioe.2009.01.004

[B22] Palonen H Tjerneld F Zacchi G Tenkanen M Adsorption of *Trichoderma reesei* CBH I and EG II and their catalytic domains on steam pretreated softwood and isolated lignin J Biotechnol 2004 107 65 72 10.1016/j.jbiotec.2003.09.011 14687972

[B23] Rahikainen JL Moilanen U Nurmi-Rantala S Lappas A Koivula A Viikari L Kruus K Effect of temperature on lignin-derived inhibition studied with three structurally different cellobiohydrolases Bioresour Technol 2013 146 118 125 23920120 10.1016/j.biortech.2013.07.069

[B24] Voutilainen SP Puranen T Siika-Aho M Lappalainen A Alapuranen M Kallio J Hooman S Viikari L Vehmaanperä J Koivula A Cloning, expression, and characterization of novel thermostable family 7 cellobiohydrolases Biotechnol Bioeng 2008 101 515 528 10.1002/bit.21940 18512263

[B25] Teugjas H Väljamäe P Product inhibition of cellulases studied with 14C-labeled cellulose substrates Biotechnol Biofuels 2013 6 104 10.1186/1754-6834-6-104 23883520 PMC3726336

[B26] Kristensen JB Felby C Jørgensen H Yield-determining factors in high-solids enzymatic hydrolysis of lignocellulose Biotechnol Biofuels 2009 2 11 10.1186/1754-6834-2-11 19505292 PMC2699335

[B27] Lavenson DM Tozzi EJ Karuna N Jeoh T Powell RL McCarthy MJ The effect of mixing on the liquefaction and saccharification of cellulosic fibers Bioresour Technol 2012 111 240 247 22342045 10.1016/j.biortech.2012.01.167

[B28] Andric P Meyer AS Jensen PA Dam-Johansen K Reactor design for minimizing product inhibition during enzymatic lignocellulose hydrolysis: I. Significance and mechanism of cellobiose and glucose inhibition on cellulolytic enzymes Biotechnol Adv 2010 28 308 324 10.1016/j.biotechadv.2010.01.003 20080173

[B29] Tejirian A Xu F Inhibition of enzymatic cellulolysis by phenolic compounds Enzyme Microb Technol 2011 48 239 247 10.1016/j.enzmictec.2010.11.004 22112906

[B30] Haven MØ Jørgensen H The challenging measurement of protein in complex biomass derived samples Appl Biochem Biotechnol 2014 172 87 101 10.1007/s12010-013-0466-x 24046255

[B31] Yu AHC Lee D Saddler JN Adsorption and desorption of cellulase components during the hydrolysis of a steam-exploded birch substrate Appl Biochem Biotechnol 1995 21 203 216

[B32] Berlin A Gilkes N Kurabi A Bura R Tu M Kilburn D Saddler J Weak lignin-binding enzymes: A novel approach to improve activity of cellulases for hydrolysis of lignocellulosics Appl Biochem Biotechnol 2005 121 163 170 10.1385/ABAB:121:1-3:0163 15917596

[B33] Kostylev M Wilson D Synergistic interactions in cellulose hydrolysis Biofuels 2012 3 1 61 70 10.4155/bfs.11.150

[B34] Börjesson J Engqvist M Sipos B Tjerneld F Effect of poly(ethylene glycol) on enzymatic hydrolysis and adsorption of cellulase enzymes to pretreated lignocellulose Enzyme Microb Technol 2007 41 186 195 10.1016/j.enzmictec.2007.01.003

[B35] Gao D Chundawat SPS Sethi A Balan V Gnanakaran S Dale BE Increased enzyme binding to substrate is not necessary for more efficient cellulose hydrolysis Proc Natl Acad Sci U S A 2013 110 10922 10927 10.1073/pnas.1213426110 23784776 PMC3703979

[B36] Wu Z Lee YY Inhibition of the enzymatic hydrolysis of cellulose by ethanol Biotechnol Lett 1997 19 10 977 979 10.1023/A:1018487015129

[B37] Cheng H Jin S Effect of ethanol and yeast on cellulase activity and hydrolysis of crystalline cellulose Enzyme Microb Technol 2006 39 1430 1432 10.1016/j.enzmictec.2006.03.027

[B38] Skovgaard PA Jørgensen H Influence of high temperature and ethanol on thermostable lignocellulolytic enzymes J Ind Microbiol Biotechnol 2013 40 447 456 10.1007/s10295-013-1248-8 23483355

[B39] Hall M Rubin J Behrens SH Bommarius AS The cellulose-binding domain of cellobiohydrolase Cel7A from *Trichoderma reesei* is also a thermostabilizing domain J Biotechnol 2011 155 370 376 10.1016/j.jbiotec.2011.07.016 21807036

[B40] Sluiter A Hames B Ruiz R Scarlata C Sluiter J Templeton D Determination of Sugars, Byproducts, and Degradation Products in Liquid Fraction Process Samples NREL Technical Report: NREL/TP-510-42623 2008 Golden, CO: National Renewable Energy Laboratory

[B41] van Tilbeurgh H Loontiens FG de Bruyne CK Claeyssens M Fluorogenic and chromogenic glycosides as substrates and ligands of carbohydrases Methods Enzymol 1988 160 45 59

[B42] Jørgensen H Vibe-Pedersen J Larsen J Felby C Liquefaction of lignocellulose at high-solids concentrations Biotechnol Bioeng 2007 96 862 870 10.1002/bit.21115 16865734

[B43] Miller GL Use of dinitrosalicylic acid reagent for determination of reducing sugar Anal Chem 1959 31 3 426 428 10.1021/ac60147a030

[B44] Pakarinen A Maijala P Stoddard F Santanen A Kymäläinen M Tuomainen P Viikari L Evaluation of annual bioenergy crops in the boreal zone for biogas and ethanol production Biomass Bioenergy 2011 35 3071 3078 10.1016/j.biombioe.2011.04.022

[B45] Kristensen JB Felby C Jørgensen H Determining yields in high solids enzymatic hydrolysis of biomass Appl Biochem Biotechnol 2009 156 127 132 10.1007/s12010-008-8375-0 18836690

[B46] Lowry OH Rosebrough NJ Farr AL Randall RJ Protein measurement with the Folin phenol reagent J Biol Chem 1951 193 265 275 14907713

[B47] Zhu Z Sathitsuksanoh N Zhang YHP Direct quantitative determination of adsorbed cellulase on lignocellulosic biomass with its application to study cellulase desorption for potential recycling Analyst 2009 134 11 2267 2272 10.1039/b906065k 19838414

